# Initial real-world experience of clazosentan for subarachnoid hemorrhage in Japan

**DOI:** 10.1016/j.wnsx.2023.100253

**Published:** 2023-12-05

**Authors:** Takuma Maeda, Mai Okawara, Manabu Osakabe, Hiroyuki Yamaguchi, Takahiro Maeda, Hiroki Kurita

**Affiliations:** a Department of Cerebrovascular Surgery, Saitama Medical University International Medical Center, Hidaka, Japan; b Department of Neurosurgery, Ohkawara Neurosurgical Hospital, Muroran, Japan

**Keywords:** Cerebral vasospasm (CVS), Clazosentan, Elderly patients, PIVLAZ, Subarachnoid hemorrhage (SAH)

## Abstract

**BACKGROUND:**

Cerebral vasospasm (CVS) is one of the most critical factors associated with clinical outcomes of patients with subarachnoid hemorrhage (SAH). Clazosentan has been investigated worldwide as a prophylactic agent to prevent CVS. We evaluated a new CVS management protocol which included clazosentan.

**METHODS:**

Consecutive 138 patients with SAH, hospitalized in our institution between January 2017 and December 2022, were included in this study. Baseline characteristics, clinical findings, and operative records were analyzed retrospectively. From May 2022, 10 mg/h clazosentan was co-administered with fasudil to all patients according to the indication in the Japanese label. Patients admitted before this date received the conventional combined protocol using the fasudil hydrochloride, nicardipine, and ozagrel.

**RESULTS:**

Eighteen (13.0%) patients received the new protocol during the CVS period (defined as day 1 up to day 14 after SAH onset). There were 54 (39.1%) elderly patients aged 75 years or older. Seventy-two (52.2%) patients underwent neurosurgical clipping, whereas 55 (39.9%) patients received endovascular coiling. Among the patients with new protocol, only one patient (5.6%) had symptomatic CVS, compared with 18 patients (15.0%) in those with conventional protocol. More patients who received the new protocol had fluid retention compared with control group (38.9% [7/18] vs. 8.3% [10/120]). Other results did not differ between the two groups.

**CONCLUSIONS:**

Clinical outcomes of the new protocol were comparable to those of conventional protocol. Clazosentan may simplify anti-vasospasm treatment. Fluid retention was a specific side-effect of clazosentan, which requires attention especially in the first half of the CVS period.

## 1 Introduction

Cerebral vasospasm (CVS) is one of the most critical factors associated with poor clinical outcomes in patients with subarachnoid hemorrhage (SAH). Approximately half of patients with CVS will develop cerebral infarction.^1^ Before the introduction of clazosentan (PIVLAZ™) in Japan, there were no effective drugs for prevention of CVS, although many drugs such as fasudil hydrochloride and ozagrel have been widely used and reported.^2^^,^^3^ Clazosentan, a selective endothelin (ET) A receptor antagonist, has been investigated worldwide as a prophylactic agent to prevent CVS and its ischemic complications after SAH.^4^ ET-1 is primarily secreted by endothelial cells and is a powerful vasoconstrictor involved in the development of CVS.^5^ Clazosentan was approved in Japan for use in the prevention of CVS in early 2022, following results from phase III trials.^6^

Until the approval of clazosentan in Japan, CVS was managed with the conventional combined protocol using fasudil hydrochloride, nicardipine, and ozagrel. Since its approval, a new simple protocol with clazosentan has been used for all eligible patients to prevent CVS. We present the clinical outcomes for patients with SAH in our institution treated with a new protocol with clazosentan and fasudil hydrochloride, compared with conventional protocol. The aim of this study was to investigate the efficacy and safety of the new protocol and refine the management and prevention of CVS.

## 2 Material and methods

**Fig. 1:**
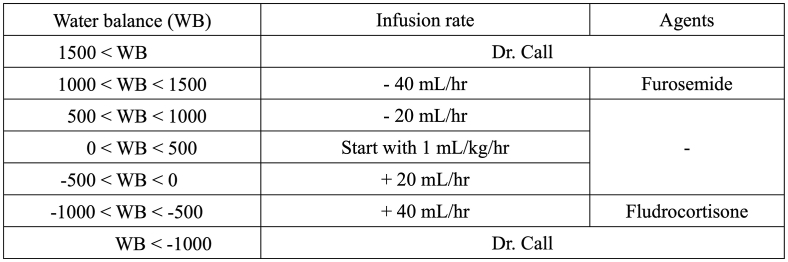
Protocol for the infusion of extracellular fluids and agents.

This study was approved by the ethics committee of the Ohkawara Neurosurgical Hospital (O2022-001). We retrospectively reviewed the medical records of all adult patients (n=138) with SAH admitted to our hospital between January 2017 and December 2022. The first day of SAH onset was defined as day 0 and the CVS period was defined as day 1 up to day 14. Until April 2022, the following conventional protocol was used in all patients. Patients received extracellular fluids (at least 2000 mL/day) to maintain mild hypervolemia. Patients received 90 mg/day of intravenous fasudil hydrochloride, continuous intravenous infusion of 1–2 mg/h nicardipine, and 80 mg/day intravenous ozagrel during the CVS period. Cilostazol (200 mg) was given orally or in a gastric tube until at least day 14 if there were no hemorrhagic complications. Since May 2022, the following simple protocol was applied to all patients according to the indication in the Japanese labels. The infusion rate of extracellular fluids was limited to 1 mL/kg/hr and adjusted based on the water balance (WB) per 24 h to maintain normovolemia (Fig. 1). Patients who had positive WB of 1000 mL/day or more received 10 mg intravenous furosemide. Just two agents against CVS were administered to all patients: 10 mg/h intravenous clazosentan and 90 mg/day intravenous fasudil hydrochloride. The following factors were compared between the new and conventional protocols: the patients’ age, sex, vascular risk factors (hypertension, diabetes mellitus, and hyperlipidemia), World Federation of Neurological Societies (WFNS) grade, aneurysmal location, therapeutic modality, symptomatic CVS, shunt dependence, surgical complications (aneurysm rupture, postoperative acute infarction, thromboembolic problem, severe contusion, coil migration, new neurological deficit, hemorrhage, and wound infection), clinical complications, and modified Rankin Scale (mRS) score at 3 months. We measured the body weight (BW) on day 1 and day 14. We also assessed the WB and urine volume per 24 h of all patients during the CVS period.

Patients were categorized based on their WFNS grade at admission: fair (grade I-III) and poor (grade IV or V). Patients were also divided into two groups based on their clinical outcome, defined as favorable (0–3) or unfavorable (4–6) according to the mRS score at 3 months. Aneurysm location was defined as the internal carotid artery (ICA), the middle cerebral artery (MCA), the anterior cerebral artery, the anterior communicating artery, or the vertebrobasilar artery. All cases of SAH were detected using a computed tomography (CT) scan. Patients also underwent a CT angiography or digital subtraction angiography to diagnose ruptured cerebral aneurysms and to investigate their detailed morphology. After this investigation, neurosurgeons and neuroendovascular physicians discussed and identified the most suitable therapeutic modality for each patient.

Neurosurgical clipping was performed under general anesthesia. Patients with endovascular coiling were treated under general anesthesia or deep sedation (defined by achievement of modified Richmond Agitation–Sedation Scale <4). Dual antiplatelet therapy was started with loading dose just before the endovascular procedures to prevent thromboembolic complications. After measuring baseline activated clotting time (ACT), the patients were administered 2000–3000 units of heparin to achieve an ACT greater than 250 s.

Routine MRI was performed at least three times, or more if required during the CVS period. If diagnosis was not possible after MRI, CT angiography, digital subtraction angiography, and perfusion imaging were also performed. As reported by Jennifer et al,^7^ we defined symptomatic CVS as the development of new focal neurological deficits or deterioration in level of consciousness caused by ischemia attributable to CVS after other possible causes had been excluded (e.g., hydrocephalus, seizure, metabolic disorder, fever, or sedation).

## 3 Statistical analysis

Quantitative variables are expressed as mean ± standard deviation. The chi-square or Fisher's exact test were used to identify covariates that could be used as binary categorical dependent variables. Unpaired sample tests using Welch's correction were used for parametric data, and Mann–Whitney *U* tests were used for nonparametric data. Statistical significance was set to *P*<0.05. SPSS version 24 (IBM Corp., Armonk, New York, USA) was used for all statistical analyses.

## 4 Results

One hundred and thirty-eight patients with a median age of 71 years were enrolled in this study. There were more female (76.1%) than male patients. Of all patients, 18 patients (13.0%) received the new protocol in the CVS period. The baseline characteristics of all patients are shown in Table 1. The most common locations of the aneurysm were the ICA and MCA (26.1% and 26.1% of all patients, respectively). A total of 72 patients (52.2%) underwent neurosurgical clipping, and 55 patients (39.9%) underwent endovascular coiling. The other 11 (8.0%) patients did not undergo a surgical procedure due to their severe general condition. Eight patients (44.4%) who were treated with the new protocol and 64 patients (53.3%) with the conventional protocol underwent neurosurgical clipping. On admission, six (33.3%) and 40 (33.3%) patients had poor WFNS grade in the patients treated with new and conventional protocols, respectively.

**Table 1:** Characteristics of patients with new and conventional protocols.

	All	New protocol	Conventional protocol	*P* value
No. of patients	138 (100)	18 (13.0)	120 (87.0)	
Median age, years (IQR)	71 (57.3, 79.0)	74 (54.5, 85.5)	71.0 (57.8, 78.0)	0.291
Age ≥75 years	54 (39.1)	8 (44.4)	46 (38.3)	0.616
Female sex	105 (76.1)	13 (72.2)	92 (76.7)	0.768
Vascular risk factors	69 (50.0)	8 (44.4)	61 (50.8)	0.801
Therapeutic modality				0.309
Surgical clipping	72 (52.2)	8 (44.4)	64 (53.3)	
Endovascular coiling	55 (39.9)	10 (55.6)	45 (37.5)	
WFNS grade				1.000
Fair (I-III)	92 (66.7)	12 (66.7)	80 (66.7)	
Poor (IV–V)	46 (33.3)	6 (33.3)	40 (33.3)	
Thick SAH (Fisher group 3)	90 (65.2)	15 (83.3)	75 (62.5)	0.112
Intracerebral hemorrhage	33 (23.9)	5 (27.8)	28 (23.3)	0.768
Location of aneurysm				
ICA	36 (26.1)	3 (16.7)	33 (27.5)	0.402
MCA	36 (26.1)	5 (27.8)	31 (25.8)	1.000
ACA	13 (9.4)	1 (5.6)	12 (10.0)	1.000
AcomA	37 (26.8)	6 (33.3)	31 (25.8)	0.570
VBA	16 (11.6)	3 (16.7)	13 (10.8)	0.440

Values are number (%) except where indicated otherwise. ACA, anterior cerebral artery; AcomA, anterior communicating artery; ICA, internal carotid artery; IQR, interquartile range; MCA, middle cerebral artery; SAH, subarachnoid hemorrhage; VBA, vertebrobasilar artery; WFNS, World Federation of Neurological Societies.

**Fig. 2:**
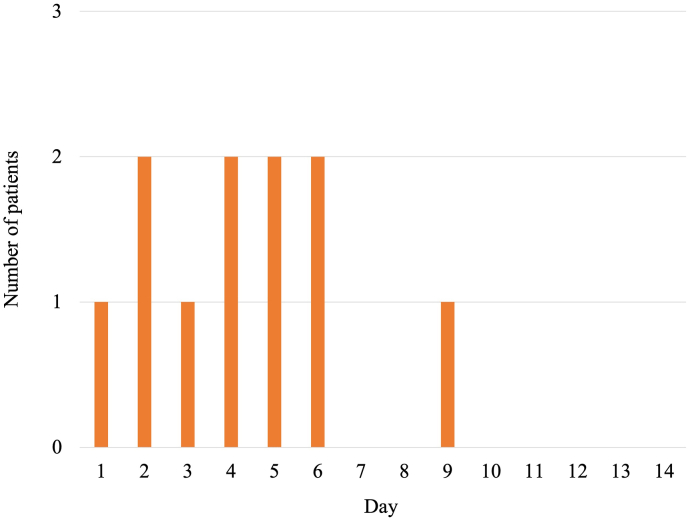
The total number of fluid retention events in the new protocol for each day during the cerebral vasospasm period. It occurs especially in the first half of the period.

The clinical outcomes in each group are shown in Table 2. In the patients treated with the new protocol, only one patient (5.6%) had symptomatic CVS, compared with 18 patients (15.0%) in those treated with the conventional protocol (*P*=0.467). Similarly, one (5.6%) patient who received the new protocol and 15 (12.5%) patients treated with the conventional protocol experienced cerebral infarction after CVS (*P*=0.465). There were significant differences in fluid retention between the treatment groups, including pulmonary edema, pleural effusion, and facial edema. A higher proportion of patients treated with the new protocol (n=7, 38.9%) had fluid retention compared with those treated with the conventional protocol (n=10, 8.3%) (*P=0.002*). Fig. 2 shows the total number of fluid retention events in the new protocol for each day during the CVS period. In one case with the new protocol, intubation was required due to pulmonary edema, although the patient fully recovered without deviating from the protocol. Other results, including surgical complications, shunt dependence, and mRS at 3 months, did not differ between the two groups.

**Table 2:** Clinical outcomes of patients with new and conventional protocols.

	New protocol (n=18)	Conventional protocol (n=120)	*P* value
Fluid retention	7 (38.9)	10 (8.3)	0.002
Pulmonary edema	2 (11.1)	2 (1.7)	0.083
Pleural effusion	5 (27.8)	7 (5.8)	0.010
Facial edema	4 (22.2)	2 (1.7)	0.003
Median weight gain, kg (IQR)	2.3 (1.175, 4.475)	−1.7 (−3.000, 0.400)	0.006
Symptomatic vasospasm	1 (5.6)	18 (15.0)	0.467
Infarction after vasospasm	1 (5.6)	15 (12.5)	0.465
Surgical complication	6 (33.3)	40 (33.3)	1.000
Rebleeding	0 (0)	2 (1.7)	1.000
Shunt dependence	3 (16.7)	34 (28.3)	0.398
mRS score at 3 months			0.792
Favorable (0–3)	11 (61.1)	79 (65.8)	
Unfavorable (4–6)	7 (38.9)	41 (34.2)	

Values are number (%) except where indicated otherwise. IQR, interquartile range; mRS, modified Rankin Scale.

**Fig. 3:**
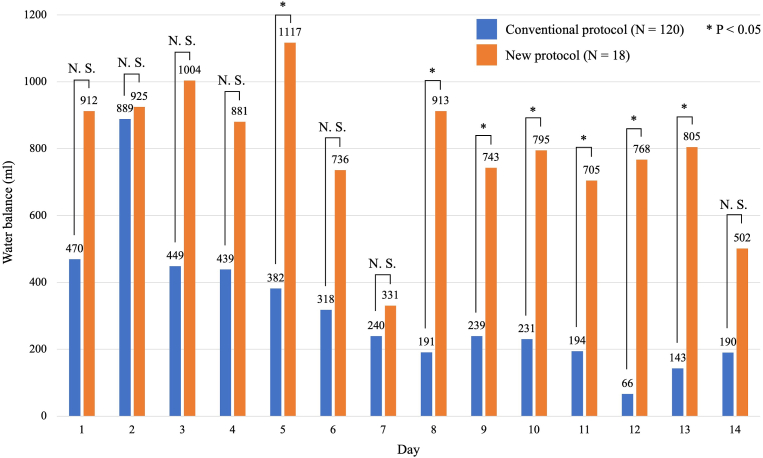
Water balance of all patients during the cerebral vasospasm period.

**Fig. 4:**
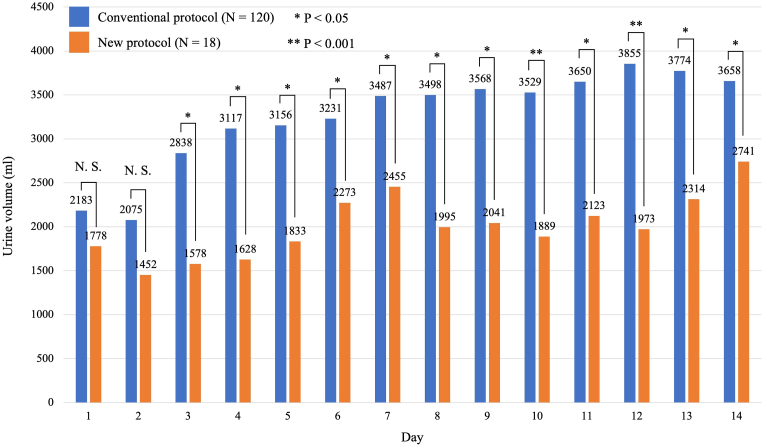
Urine volume of all patients during the cerebral vasospasm period.

We investigated the WB and urine volume per 24 h of all patients during the CVS period (see Fig. 3, Fig. 4). There were significant differences in WB on days 5, 8, 9, 10, 11, 12, and 13 between the groups (*P*<0.05). Patients treated with the new protocol showed more positive WB trends through the CVS period. Moreover, the urine volume was significantly lower in patients treated with the new protocol compared with those receiving the conventional protocol, except for the first two days (Fig. 4). Finally, we measured the BW of patients on day 1 and day 14 (Table 2). The median weight change was greater in patients treated with new protocol (2.3 kg) than those with conventional protocol (−1.7 kg) (*P*=0.006). In other words, patients treated with the new protocol including clazosentan and fasudil hydrochloride gained weight, whereas those treated with the conventional protocol lost weight.

Subgroup analyses investigating the elderly (aged 75 years or older) and younger groups were performed (Table 3). In the elderly group, there were significant differences in fluid retention (*P*<0.001) and median weight gain (*P*=0.040) between the new (n=8) and conventional (n=46) protocols. In contrast, there was no significant difference in any variables, including fluid retention, in the younger group.

**Table 3:** Subgroup analysis of clinical outcomes in elderly and younger groups.

	Elderly group (age ≥75 years)			Younger group		
	New protocol	Conventional protocol	*P* value	New protocol	Conventional protocol	*P* value
No. of patients	8 (14.8)	46 (85.2)		10 (11.9)	74 (88.1)	
Poor WFNS grade (IV–V)	4 (50.0)	20 (43.5)	1.000	2 (20.0)	20 (27.0)	1.000
Fluid retention	6 (75.0)	2 (4.3)	<0.001	1 (10.0)	8 (11.6)	1.000
Pulmonary edema	2 (25.0)	0 (0)	0.020	0 (0)	2 (2.7)	1.000
Pleural effusion	4 (50.0)	2 (4.3)	0.003	1 (10.0)	5 (7.2)	0.544
Facial edema	3 (37.5)	1 (2.3)	0.008	1 (10.0)	1 (1.4)	0.225
Median weight gain, kg (IQR)	3.20 (1.18, 4.90)	−0.90 (−2.58, 0.98)	0.040	2.30 (0.60, 3.05)	−2.00 (−3.10, 3.05)	0.127
Symptomatic vasospasm	1 (12.5)	7 (15.2)	1.000	0 (0)	11 (14.9)	0.346
Infarction after vasospasm	1 (12.5)	5 (13.5)	1.000	0 (0)	10 (14.1)	0.349
Surgical complication	2 (25.0)	13 (28.3)	1.000	4 (40.0)	27 (36.5)	1.000
Rebleeding	0 (0)	2 (4.3)	1.000	0 (0)	0 (0)	1.000
Shunt dependence	2 (25.0)	18 (39.1)	0.695	1 (10.0)	16 (21.6)	0.679
Favorable outcome (mRS score 0–3)	3 (37.5)	20 (43.5)	1.000	8 (80.0)	59 (79.7)	1.000

Values are number (%) except where indicated otherwise. IQR, interquartile range; mRS, modified Rankin Scale; WFNS, World Federation of Neurological Societies.

## 5 Discussion

During the past 20 years up to 2019, registry research throughout Japan shows that clinical outcomes for patients with SAH had not improved.^8^ Moreover, non-adjusted incidence of SAH has been rising with the aging of the Japanese population.^9^ CVS has been identified as one of the stronger factors associated with poor clinical outcome of patients with SAH.^10^ Thus, prevention of CVS is expected to lead to improved clinical outcomes of patients with SAH.

The release of oxyhemoglobin from red blood cells generates the release of ET-1, a powerful and long-lasting vasoconstrictor, mainly from endothelial cells, leading to development of CVS.^11^^,^^12^ ET receptor antagonists are reported to reduce the incidence of CVS.^13^ In particular, clazosentan is a selective ETA receptor antagonist, and has been shown to reduce CVS after experimental SAH.^14^ Clazosentan was approved in Japan in early 2022, for the first time worldwide.^4^ The approval was supported by the results from the JapicCTI163369 and JapicCTI163368 phase III trials.^6^ Although there have been many studies and clinical trials providing experimental data,^6^^,^^15^ there are no reports on actual clinical use in practice after approval at the time of early 2023. Moreover, this study enrolled many patients aged 75 years or older, who were excluded from the phase III trials. Although in a limited number of patients, this study is the early description of the clinical effects and specific side effects of clazosentan in combination with fasudil hydrochloride, which was forbidden in the phase III trials due to the risk for excessive hypotension, and it was only permitted as an intra-arterial rescue administration. In the previous double-blind trials,^6^^,^^16^ clazosentan significantly reduced the incidence of angiographic CVS from 52.3% to 26.6% (*P*=0.0001) and fasudil hydrochloride from 61% to 38% (*P*=0.0023). Although head-to-head comparisons are challenging due to differences in clinical settings and treatment approaches, both interventions demonstrated notable reductions in the incidence of angiographic CVS and poor clinical outcomes compared to placebo. There is currently a lack of clinical trials directly comparing clazosentan and fasudil hydrochloride, as well as the combination of these agents. Given the limited number of patients in this study, it is not possible to draw conclusions regarding combination therapy. However, a low incidence of CVS was observed in the 18 patients treated with both drugs, suggesting a potentially favorable effect on CVS control. Further validation of clazosentan monotherapy and combination therapy is needed.

In this study, the incidence of symptomatic CVS was only 5.6% with the new protocol including clazosentan, which is a low frequency compared with previous reports (30–70%).^17^ The incidence of CVS in the new protocol was lower than those in conventional protocol, although no significant difference was observed. The difference may become statistically significant as the number of cases increases in the near future. The clinical outcomes of patients treated with this simple protocol were comparable with conventional combined protocol. The median age of patients treated with the new protocol was non-significantly higher than in the control group. Older patients, combined with small sample size in the new protocol, may have resulted in non-superiority. Additionally, this is the early phase study of a protocol with clazosentan in combination with fasudil hydrochloride in a real-world setting after approval. The more this protocol is refined with more subjects, the more overall clinical outcomes of patients with SAH may improve.

With the new protocol, patients were more likely to have fluid retention than those treated with the conventional protocol. However, there were no cases of protocol deviation with the new protocol. Notably, patients treated with the new protocol experienced decreased urine volume and a positive trend in WB, resulting in weight gain in this study. Fluid retention is a common adverse effect of ETA receptor antagonists.^18^ Previous studies have suggested that, of the two ET receptor subtypes, ETB receptors should not be blocked because they are involved in natriuresis and diuresis.^5^^,^^19^ In contrast to these suggestions, clinical data revealed that patients treated with ETA receptor antagonists had greater risk of fluid retention than those treated with dual ET receptor antagonists.^20^^,^^21^ Administration of a selective ETA receptor antagonist led to significant reduction in blood pressure, plasma volume expansion, and a greater increase in vascular permeability than administration of a non-selective antagonist in rats.^18^ Isolated vessel experiments of rats showed that administration of a selective ETA antagonist increased vascular permeability via ETB receptor overstimulation as ET-1 which could not bind to the ETA receptor led to overstimulation of the unblocked ETB receptor. Moreover, administration of a selective ETA receptor antagonist increased antidiuretic hormones such as norepinephrine, vasopressin, and aldosterone compared to dual ET receptor antagonists.^18^ These results may explain the cause of fluid retention in this study in patients treated with the new protocol including clazosentan. In addition, the rates of pleural effusion and facial edema with the new protocol are higher compared to the rates observed in phase III trials. The fasudil hydrochloride also has vasodilator effects on pulmonary artery, although the association with pleural effusion is not clear.^22^ The use of clazosentan co-administrated with fasudil hydrochloride may have led to the differences in this study compared with phase III trials.

In clinical practice, management of WB with attention to fluid retention is important. In the new protocol, we limited the infusion rate of extracellular fluids to 1 mL/kg/hr, less than previously standard.^23^ Additionally, early diuretic administration was given even in the CVS period. Despite these controls, there was a more positive WB trend leading to fluid retention in the new protocol compared with the conventional protocol. However, with careful management, clinical outcomes were comparable with the conventional combined protocol. We should pay attention to early diagnosis and treatment of pulmonary edema and pleural effusion. As shown in this study, this adverse event should be especially considered in elderly patients.

In our new protocol, only clazosentan and fasudil hydrochloride were administered as anti-vasospasm agents. In previous studies, various agents such as fasudil hydrochloride, ozagrel, nimodipine, nicardipine, and cilostazol were used and investigated.^24^ However, there is no apparent consensus to date. It is important to simplify anti-vasospasm treatment, considering standardization and medical economics. Our protocol is very simple due to the small number of agents and no need to adjust the dose based on age or BW. Additionally, our protocol did not require antiplatelet therapy, which can lead to excessive bleeding. Clinical outcomes with the new protocol were comparable, thus, the advantage of clazosentan revealed in this initial experience was the simplified management and prevention of CVS.

## 6 Limitations

We acknowledge several limitations associated with the study. First, this study is retrospective and from a single institution. Second, the number of enrolled patients with the new protocol was limited (18 of 138 patients), and this contributes to a lack of statistical power. Specifically, this small number of patients with the new protocol is insufficient for an analysis of outcomes. In addition, the imbalance between groups in the therapeutic modality and also in the rate of patients with Fisher group 3. Despite these limitations, this study reports the early real-world experience with this new drug for the prevention of CVS and appears to be a pivotal publication including adverse events. In particular, it is important to have knowledge of fluid retention before the drug is approved in other countries. It is noteworthy that patients treated with the new protocol showed comparable clinical outcomes despite the discontinuation of all conventional concomitant therapies. Therefore, the current real-world advantage lies in the simplification of treatment and its potential contribution to medical economics. Further studies are warranted to assess the specific impact of clazosentan in isolation, without fasudil hydrochloride.

## 7 Conclusions

Clinical outcomes of patients treated with the new protocol for CVS with clazosentan co-administered with fasudil hydrochloride were comparable to those with the conventional combined protocol. Clazosentan may simplify the anti-vasospasm treatment. Fluid retention was a specific complication of clazosentan, which requires attention especially in the first half of the CVS period. Assessment of the long-term outcomes of patients who have received clazosentan in the real world needs more subjects.

## Funding

This research did not receive any specific grant from funding agencies in the public, commercial, or not-for-profit sectors.

## CRediT authorship contribution statement

**Takuma Maeda:** Methodology, Writing - original draft, Conceptualization. **Mai Okawara:** Supervision. **Manabu Osakabe:** Writing - review & editing. **Hiroyuki Yamaguchi:** Writing - review & editing. **Takahiro Maeda:** Writing - review & editing. **Hiroki Kurita:** Writing - review & editing, Project administration.

## Declaration of competing interest

The authors declare that they have no known competing financial interests or personal relationships that could have appeared to influence the work reported in this paper.
